# Acute ST-Elevation Myocardial Infarction (STEMI) Diagnoses in a Biventricular-Paced ECG

**DOI:** 10.7759/cureus.96210

**Published:** 2025-11-06

**Authors:** Dhruva Govil, Ziad R Affas, Chad Nicholson, Christopher Bradley, Marcel Zughaib

**Affiliations:** 1 Internal Medicine, Henry Ford Providence Hospital, Southfield, USA; 2 Internal Medicine, Henry Ford Macomb hospital, Clinton township, USA; 3 Cardiology, Henry Ford Health System, Southfield, USA; 4 Cardiology, Henry Ford Providence Hospital, Southfield, USA

**Keywords:** acute coronary syndrome, biventricular (biv), biv icd, biv pacing, inferior stemi, sgarbossa criteria

## Abstract

Diagnosing acute ST-elevation myocardial infarction (STEMI) in patients with ventricular pacing is difficult due to abnormal activation patterns that can mask ischemic changes on ECG. Unlike traditional right ventricular pacing, biventricular (BiV) pacing produces a better physiologic depolarization, improving detection. A 69-year-old male with heart failure with reduced ejection fraction and a BiV pacemaker presented with acute chest pain. ECG revealed inferior STEMI, and successful treatment was done for a critically stenotic proximal right coronary artery. BiV pacing provides a more synchronous and physiologic ventricular activation pattern compared to other modalities that disrupt normal depolarization and hinder ECG interpretation with acute ischemia. Within this case, the physiologic electrical activation produced by BiV pacing allowed for accurate ECG identification of STEMI and improved mechanical synchrony. BiV pacing can enhance ECG detection of acute STEMI compared to traditional pacing, aiding prompt diagnosis and intervention in patients with heart failure.

## Introduction

Ventricular pacing electrocardiogram (ECG) interpretation is a significant diagnostic challenge, particularly in the context of suspected ST-elevation myocardial infarction (STEMI). Conventional ventricular pacing, especially from the right ventricle (RV), produces non-physiologic depolarization patterns that can obscure or mimic ischemic changes, complicating accurate diagnosis. However, biventricular (BiV) pacing or cardiac resynchronization therapy more closely replicates native conduction by pacing both ventricles simultaneously, potentially preserving some ECG sensitivity to acute ischemic events [1,2].

Unlike RV pacing, BiV pacing produces a narrower QRS and improved ventricular contraction, which may help preserve ischemia-related ECG changes. Still, little is known about how ST-segment changes appear in BiV-paced rhythms [3]. Exciting diagnostic frameworks, such as the original and modified Sgarbossa criteria, have improved ischemic ECG interpretation in left bundle branch block and RV pacing, though BiV-paced rhythms remain unclear. So far, only a handful of case reports have shown that STEMI can be detected in this setting, and no standardized criteria currently exist to guide ECG interpretation in these patients [3,4,5].

## Case presentation

A 69-year-old male presented with two hours of acute-onset chest pain. He reported having intermittent, milder chest discomfort over the past several months, but described this pain as more intense and qualitatively different. On arrival, he was hemodynamically stable. The physical exam was unremarkable; heart sounds were normal with no murmurs or gallops.

**Figure 1:**
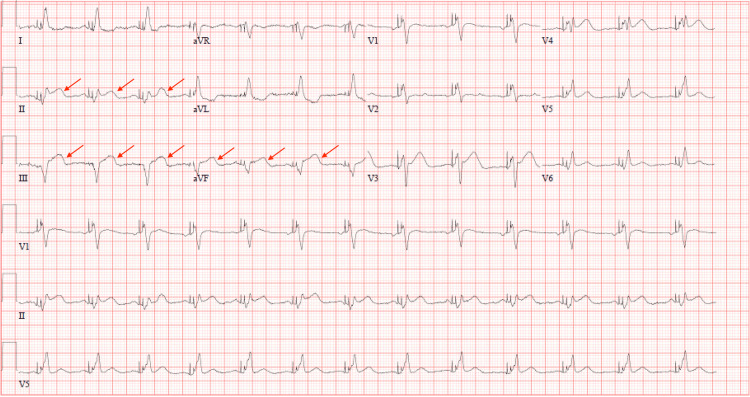
Initial ECG upon arrival Concordant ST elevation in Lead II and V4 suggestive of STEMI meeting Sgarbossa 1 criteria [1].

**Figure 2:**
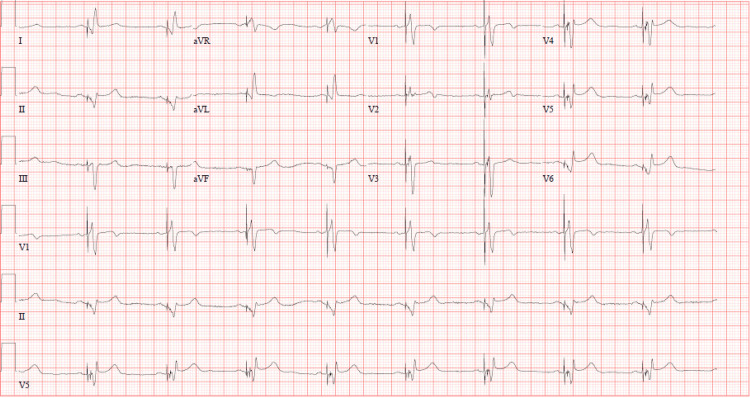
Prior to presentation, myocardial infarction ECG

The patient had a history of heart failure with reduced ejection fraction of non-ischemic origin and non-obstructive coronary artery disease. Due to evidence of electrical dyssynchrony with left bundle branch block (LBBB), he had previously undergone implantation of a biventricular implantable cardioverter-defibrillator (BiV ICD) for cardiac resynchronization. The initial differential diagnosis included acute coronary syndrome (ACS), pericarditis, and pulmonary embolism. Initial ECG revealed ST-segment elevations in the inferior leads compared with the prior baseline ECG, consistent with a new ischemic event (Figures 1-2). Although paced-rhythm interpretation of ischemic changes is often limited, the degree and morphology of the ST elevation in this BiV-paced rhythm raised clinical concern. High-sensitivity troponin was elevated. Based on these findings and the clinical presentation, the cardiac catheterization lab was activated emergently.

**Figure 3:**
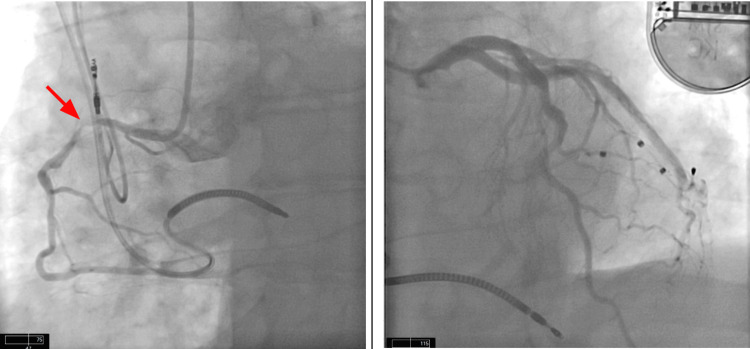
Cardiac Catheterization Left: right coronary artery (RCA) with 95% Stenosis in the proximal RCA. Right: Left coronary artery without any significant disease

**Figure 4:**
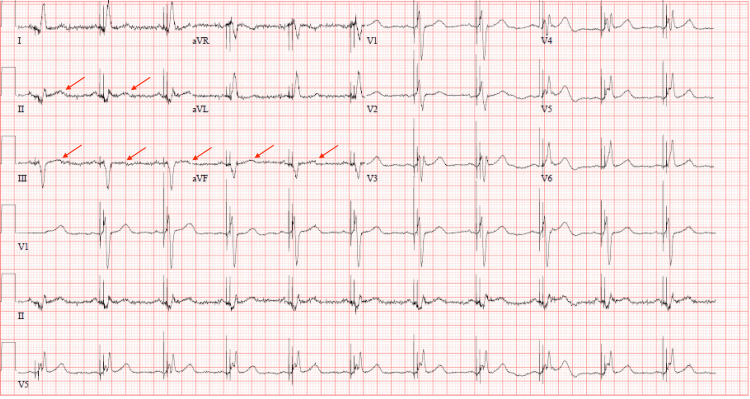
Post-catheterization ECG There seems to be an improvement in the ST elevation.

Coronary angiography revealed a critical 95% stenosis of the proximal right coronary artery. A successful percutaneous coronary intervention was performed using two overlapping drug-eluting stents. The procedure restored thrombolysis in myocardial infarction (TIMI-3) flow and eliminated residual stenosis (Figure 3). Post-procedure ECG showed resolution of ST-segment elevation (Figure 4). The patient remained clinically stable throughout his hospitalization and reported no further chest pain at his two-week follow-up.

## Discussion

This case underscores an important clinical observation: in contrast to RV pacing or LBBB, BiV pacing can permit detection of acute ischemic ECG changes due to its more physiologic pattern of ventricular activation [1,3,4]. Cardiac resynchronization therapy (CRT) typically paces the right and left ventricles, resulting in improved synchronous depolarization that closely resembles native conduction [6]. This may reduce the degree of QRS distortion and thus increase the visibility of superimposed ischemic ST-segment shifts.

In normal RV pacing or intrinsic LBBB, the abnormal depolarization and repolarization vectors make ischemic changes difficult to distinguish, often leading to false negatives or diagnostic delay in suspected STEMI [3,7]. This issue has been well documented, with numerous cases of delayed or missed diagnosis in paced rhythms due to over-reliance on standard ECG interpretation algorithms developed for non-paced hearts.

Although scoring systems like the original and modified Sgarbossa criteria were developed to address this issue, these tools were validated primarily in patients with LBBB or RV pacing, not in those with BiV pacing [3,7]. Given the different vector patterns and relative synchronization BiV pacing offers, these criteria may either under- or overestimate ischemic burden in this population [1].

Importantly, this case illustrates that careful ECG interpretation, particularly with attention to lead concordance, ST-segment trends, and comparison with prior tracings, remains a powerful diagnostic tool even in the setting of paced rhythms [2,4,8]. It also suggests that BiV pacing not only creates mechanical synchrony but also has a diagnostic advantage in ACS, by preserving the ability to detect ST-segment elevation. This is especially important since most patients with heart failure are treated with CRT, many of whom have an increased risk for cardiovascular events [6]. Improved ECG interpretability could lead to faster STEMI recognition and improved outcomes.

As conduction system pacing strategies such as left bundle branch pacing (LBBP) and His-bundle pacing (HBP) become more common, clinicians should remain vigilant about unique ECG signatures. These newer pacing modalities may offer similar diagnostic benefits to BiV pacing in preserving ischemic ECG interpretation, though further research is needed [9].

## Conclusions

This case highlights the need to rethink how we interpret ECGs in patients with advanced pacing systems. BiV pacing may offer a diagnostic advantage for ACS by improving physiologic depolarization patterns, allowing for better identification of ischemic changes. As CRT becomes more widespread, further research is needed to develop and validate STEMI criteria specific to BiV-paced rhythms. Until then, clinicians should maintain a high index of suspicion and use a multimodal approach, including serial ECGs, biomarkers, bedside echocardiography, and clinical context, to guide urgent decision-making in these patients.
